# Improved correction formulas to estimate iohexol clearance from simple models

**DOI:** 10.1007/s00228-023-03535-y

**Published:** 2023-07-08

**Authors:** Qian Dong, Uwe Fuhr, Elke Schaeffner, Markus van der Giet, Natalie Ebert, Max Taubert

**Affiliations:** 1 Department I of Pharmacology, Center for Pharmacology, Faculty of Medicine and University Hospital Cologne, University of Cologne, Gleueler Straße 24, Cologne, 50931 Germany; 2 Institute of Public Health, Charité Universitätsmedizin Berlin, Berlin, Germany; 3 Department of Nephrology, Charité Universitätsmedizin Berlin, Berlin, Germany

**Keywords:** Correction formula, Iohexol plasma clearance, Measured glomerular filtration rate, Three-compartment model

To the editor,

Measuring glomerular filtration rate by iohexol plasma clearance has become a preferred method in clinical practice and research [1–9], but ambiguities remain regarding the choice of pharmacokinetic modeling when evaluating iohexol concentration. We recently showed that a three-compartment pharmacokinetic model provides better iohexol clearance estimates than the two-compartment approach in elderly subjects in the Berlin Initiative Study (BIS) [7, 8]. However, implementation of the presented model in clinical and research practice is complicated by the required technical expertise and the complexity of Bayesian software [7]. A viable alternative could be the use of correction formulas which rely on traditional one- or two-compartment models and correct the obtained clearance estimates for the bias introduced by the omission of compartments [9–14]. Well-known examples for established correction formulas are the Bröchner-Mortensen (BM) equation [12] and the further simplified Chantler (Ch) formula [14] based on a one-compartment model, which have recently been shown to perform well in iohexol data from the BIS [9]. Such an evaluation remains to be carried out based on a three-compartment model, which is the subject of this letter.

Data from the BIS, including 546 individuals with data obtained up to 300 min post-injection, were evaluated [7, 8]. One-, two-, and three-compartment models were used to estimate iohexol clearance (CL1, CL2, and CL3), respectively. CL1 was estimated based on the slow component of iohexol concentrations 120 to 300 min post-dose, CL2 was estimated based on the protocol by Schwartz et al. [11] as carried out in the BIS [8], and CL3 was estimated using the empirical Bayes approach based on the three-compartment model [7]. Equations resembling the BM and Ch formulas were then fitted to correct CL1 and CL2 results, using CL3 as the reference (Table 1) [12, 14]. A leave-one-out cross-validation [15] was utilized to assess the bias and root mean squared error (RMSE), as well as to evaluate Lin’s concordance correlation coefficient (CCC) [16] and the relative total deviation index (TDI) for a range of coverage probabilities (CP) [17]. A TDI ≤ 10% for a CP of 90% (TDI_90_) and an at least substantial CCC of ≥ 0.95 were considered optimal. R 4.2.1 [18] and NONMEM 7.4.2 [19] were used as statistical software.

**Table 1:** Performance of correction formulas based on leave-one-out cross-validation, comparing iohexol clearance estimates obtained from different correction formulas to reference clearance values from three-compartment model

Nr.	Equation	Bias (mL/min) mean (95% CI)	Relative bias (%) mean (95% CI)	RMSE (mL/min)	CCC	TDI_90_ (%)
1 (BM)	$\text{CL=0.872}{\times}\text{CL1-0.000560}{\times}{\text{CL1}}^{2}$	− 0.0949 (− 0.428 to 0.238)	− 0.103 (− 0.616 to 0.411)	3.97	0.974	10.6
2 (Ch)	$\text{CL=0.824}{\times}\text{CL1}$	− 0.357 (− 0.698 to − 0.016)	− 0.953 (− 1.50 to − 0.409)	4.08	0.974	11.5
3 (BM)	$\text{CL=0.894}{\times}\text{CL2+0.0000246}{\times}{\text{CL2}}^{2}$	− 0.0415 (− 0.288 to 0.205)	− 0.0331 (− 0.411 to 0.344)	2.94	0.986	7.71
4 (Ch)	$\text{CL=0.896}{\times}\text{CL2}$	− 0.0313 (− 0.277 to 0.214)	− 0.0022 (− 0.378 to 0.374)	2.92	0.986	7.68

*CL* iohexol clearance estimates obtained from different correction formulas, *CL1* one-compartment clearance estimates, *CL2* two-compartment clearance estimates, *BM* Bröchner-Mortensen–like equation, *Ch* Chantler–like equation, *CI* confidence interval, *RMSE* root mean square error, *CCC* Lin’s concordance correlation coefficient, *TDI*_*90*_ total deviation index for a coverage probability of 90%

BM- and Ch-like equations performed similarly well (Table 1), with an absolute bias < 1 mL/min, an RMSE between 2.92 and 4.08 mL/min, and a substantial concordance for all equations. The TDI_90_ goal was achieved with Eqs. 3 and 4 based on two compartments, while it was missed with Eqs. 1 and 2 based on one compartment. Differences between BM-like and Ch-like equations were negligible with equations based on two compartments, while the BM-like equation provided a notably lower absolute bias than the Ch-like equation in the case of one compartment (− 0.0949 versus − 0.357 mL/min). In summary, our evaluation demonstrates that correction formulas in conjunction with one- or two-compartment models can provide adequate clearance estimates with only a minimal loss in accuracy and precision compared to a three-compartment model. When one-compartment estimates are available, the BM-like correction formula might be a good choice, while the simple Ch-like formula might be sufficient if two-compartment estimates are available. This provides the means to efficiently estimate iohexol clearance in settings where complexities and costs associated with the implementation of a Bayesian model are prohibitive. Whether the slight loss of accuracy and precision compared to the three-compartment model is acceptable depends on the clinical context and should therefore be judged on a case-by-case basis. Further validation in diverse patient populations is required, and the evaluation of additional, potentially non-linear correction formulas might provide further improvements.

## Data Availability

The data that support the findings of this study are available from the corresponding author upon reasonable request.

## Declarations

### Disclaimer

The funder had no role in design, collection, analysis, and interpretation of data, and writing and publication of the manuscript.
